# 

**Published:** 1917-04

**Authors:** 


CONTENTS.

| | Page |
|---|---|
| Introduction to a Discussion on the Diagnosis of Tuberculosis. By LATIMER JAMES SHORT, M.D., B.S. Lond., M.D. Brist., D.P.H. Cantab. | 1 |
| Notes on Plague in Bristol in 1916. By LIEUT.-COLONEL D. S. DAVIES, R.A.M.C. | 16 |
| Chronic Appendicitis in its Relation to Dyspepsia. By H. GOODWYN, F.R.C.S. Ed. | 18 |
| Reviews of Books | 22 |
| Editorial Notes | 24 |
| New Drugs and Preparations for the Sick | 32 |
| Obituary Notices | 35 |

